# Smoking During Pregnancy Among Immigrant Women With Same-Origin and Swedish-Born Partners

**DOI:** 10.1093/ntr/ntaa145

**Published:** 2020-08-09

**Authors:** Marcelo L Urquia, Sol Juarez, Elizabeth Wall-Wieler, Anders Hjern

**Affiliations:** 1 Manitoba Centre for Health Policy, Department of Community Health Sciences, Rady Faculty of Health Sciences, University of Manitoba, Winnipeg, MB, Canada; 2 Centre for Health Equity Studies, Stockholm University/Karolinska Institute, Stockholm, Sweden; 3 Department of Public Health Sciences, Stockholm University, Stockholm, Sweden; 4 Department of Medicine, Clinical Epidemiology Division, Karolinska Institute, Stockholm, Sweden

## Abstract

**Introduction:**

Although ethnically mixed couples are on the rise in industrialized countries, their health behaviors are poorly understood. We examined the associations between partner’s birthplace, age at immigration, and smoking during pregnancy among foreign-born women.

**Methods:**

Population-based register study including all pregnancies resulting in a livebirth or stillbirth in Sweden (1991–2012) with complete information on smoking and parental country of birth. We compared the prevalence of smoking during pregnancy between women in dual same-origin foreign-born unions (*n* = 213 111) and in mixed couples (immigrant women with a Swedish-born partner) (*n* = 111 866) using logistic regression. Swedish-born couples were used as a benchmark.

**Results:**

The crude smoking rate among Swedish women whose partners were Swedish was 11%. Smoking rates of women in dual same-origin foreign-born unions varied substantially by birthplace, from 1.3% among women from Asian countries to 23.2% among those from other Nordic countries. Among immigrant groups with prevalences of pregnancy smoking higher than that of women in dual Swedish-born unions, having a Swedish-born partner was associated with lower odds of smoking (adjusted odds ratios: 0.72–0.87) but with higher odds among immigrant groups with lower prevalence (adjusted odds ratios: 1.17–5.88). These associations were stronger among women immigrating in adulthood, whose smoking rates were the lowest.

**Conclusions:**

Swedish-born partners “pull” smoking rates of immigrant women toward the level of smoking of Swedish-born women, particularly among women arrived during adulthood. Consideration of a woman’s and her partner’s ethnic background and life stage at migration may help understand smoking patterns of immigrant women.

**Implications:**

We found that having a Swedish-born partner is associated with higher rates of smoking during pregnancy among immigrants from regions where women smoke less than Swedish women, but with lower smoking rates among immigrants from regions where women smoke more. This implies that prevention efforts should concentrate on newly arrived single women from low prevalence regions, such as Africa and Asia, whereas cessation efforts may target women from high prevalence regions, such as other European countries. These findings suggest that pregnancy smoking prevention or cessation interventions may benefit from including partners and approaches culturally tailored to mixed unions.

## Introduction

Although smoking rates are declining over time, smoking remains a major threat to health in the foreseeable future. Worldwide projections estimate that there will be about 1.1 billion tobacco smokers by 2025 along with high variability in smoking initiation and cessation rates between countries.^1^ Smoking during pregnancy is of particular concern, as it is associated with maternal illness and newborn’s adverse outcomes, such as fetal and infant mortality.^2,3^

The prevalence of smoking in pregnancy also varies considerably worldwide, with rates of 0.8% in Africa to 8.1% in Europe in 2015.^4^ However, variability persists within regions. For example, among Nordic countries the prevalence varies from 4.5% in Iceland to 10.9% in Sweden to 25.2% in Denmark.^4^ International migration adds to the complexity of domestic tobacco control strategies, since immigrants may have smoking behaviors more similar to those prevalent in their countries of origin^4^ and require culturally tailored interventions.^5,6^ Health behaviors and birth outcomes of immigrants are known to be influenced by acculturation or assimilation,^7,8^ a process by which newcomers progressively fusion with locals by incorporating their beliefs, attitudes, and behaviors. Commonly used proxies of acculturation include maternal characteristics such as nativity and ethnic origin, language spoken, and time since immigration.^9^

Less attention has been devoted to paternal characteristics, particularly regarding their role in the acculturation of immigrant women.^10^ Intermarriage has been regarded as the final step in the assimilation process, as marrying a native-born represents a strong exposure to the language, customs, and networks of the host society.^11^ In contrast, same-origin partners of immigrant women may tend to reinforce premigration behaviors and buffer assimilation influences. Associations between health behaviors of immigrant women and the birthplace of their partners may differ according to age at arrival.^12^ A woman migrating during adulthood may already be married and have made decisions about smoking pre-migration, whereas a woman who arrived during childhood or adolescence may choose a partner among her peers who underwent a similar socialization, including exposure to smoking (i.e., assortative mating).

Smoking is an interpersonally transmitted behavior, and partners are key significant others that may play a powerful role in the assimilation process^11^ by transmitting either their own behaviors or the behaviors that are most common in their social context. Acculturation influences may manifest more clearly in behavioral outcomes, such as smoking, than in more distant outcomes affected by them, such as birthweight, preterm birth, or infant death.^13^ Moreover, smoking has been regarded as an informative outcome for studying acculturation.^14^

As international migration and ethnically mixed unions are on the rise,^11^ we examined the associations between partner’s birthplace and smoking during pregnancy among foreign-born women in the Swedish multicultural society. Our objective was to assess the associations between partner’s birthplace and smoking during pregnancy among foreign-born women. We further explored whether this association is modified by women’s age at arrival.

## Methods

### Study Population

This is a nationwide study conducted in Sweden, where maternity care is provided free of charge to all residents. Virtually all women give birth in hospitals, in most cases serviced by midwives. The study population included all pregnancies resulting in a live birth or stillbirth in Sweden from January 1, 1991 to December 31, 2012, which delivery records had complete information on smoking in early pregnancy and both maternal and paternal country of birth.

Ethical approval for this study was obtained from the Central Ethical Review Board of Stockholm in 2013 (decision no 2013/1058-32).

Pregnancy records were obtained from the Swedish Medical Birth Register (SMBR), which covers approximately 99% of all births that occur in Sweden.^15,16^ Using a unique personal identification number, the SMBR was linked with the Total Population Registry (for information on woman and partner’s country of origin, age, and year of arrival), the Longitudinal Integration Database for Health Insurance and Labour Market Studies (LISA, LOUISA before 2004) (for socioeconomic information), and the Multi-Generational Registry (for information on adoptees, partner’s age).

### Smoking During Pregnancy

The SMBR has collected valid information on self-reported maternal smoking at the first antenatal care visit (around 8–12 gestation weeks) since 1982. A validation study showed that self-reported smoking had high agreement with cotinine levels in maternal serum (kappa = 0.82).^17^ Ninety-five percent of self-reported nonsmokers were also nonsmokers after cotinine measurements.^17^

### Parental Birthplace

The country of origin was identified for both the mother and the father of the newborn as Swedish-born or foreign-born. Foreign-born parents were further divided into 11 categories: other Nordic Countries (Denmark, Finland, Iceland and Norway); Eastern Europe; Western Europe; Former Yugoslavia; Australia–New Zealand–United States, and Israel; Middle East; Sub-Saharan Africa; East Africa; North Africa; Asia; and Latin America.

### Data Analysis

We used stratified multiple logistic regression to compare the prevalence of smoking during pregnancy between women in dual foreign-born unions (foreign-born women with a foreign-born partner of the world same region) and women in mixed unions (foreign-born women with a Swedish-born partner) within maternal world region of birth. Swedish-born women were used as a contextual benchmark but not directly compared with immigrant women because they lack immigration-specific characteristics such as age at arrival and year of arrival to Sweden. We estimated odds ratios and adjusted prevalence of smoking for the comparison groups by computing predicted probabilities from a full model including pregnancy and sociodemographic characteristics. These included mother’s age, father’s age, disposable household income estimated for the year before birth (reported in quintiles), municipality of residence (urban/rural), and year of birth of child. Family circumstances included parental cohabitating status (yes/no), and whether this was the mother’s first child (yes/no). Among foreign-born women, their age at arrival, and year of arrival were also included (Table 1).

**Table 1.** Sociodemographic Characteristics of Pregnancies by Maternal and Paternal Birthplace, Sweden, 1991–2012

Women	Swedish	Foreign born	Foreign born
Partners	Swedish (*n* = 1 490 049)	Swedish (*n* = 111 866)	Foreign born (*n* = 213 111)
Smoking during pregnancy, %	11.6	11.0	8.9
Mother’s age, y, mean (SD)	29.7 (5.0)	30.5 (5.2)	29.0 (5.6)
Mother’s age, y			
<20	1.6	1.4	2.6
20–24	13.7	12.0	20.9
25–29	33.6	29.0	31.6
30–34	33.8	34.2	27.2
35+	17.3	23.4	17.8
Father’s age, y, mean (SD)	32.6 (5.7)	35.1 (7.4)	34.7 (7.0)
No previous births, %	43.1	44.0	34.5
Not cohabiting with father of child	4.0	5.0	8.0
Unknown	2.4	2.3	2.1
Disposable household income			
Quintile 1 (lowest)	12.2	20.6	58.4
Quintile 2	19.6	21.2	22.0
Quintile 3	22.4	18.4	9.5
Quintile 4	23.2	18.3	5.7
Quintile 5 (highest)	22.7	21.6	4.4
Unknown	0.1	N/A	N/A
Rural residence, %	16.6	11.5	5.8
Unknown	0.2	0.2	0.1
Birth year of the child			
1991–1995	21.5	18.3	15.9
1996–2000	21.0	18.0	19.3
2001–2005	22.7	23.0	21.6
2006–2012	34.0	41.0	43.2
Age at arrival to Sweden, y			
0–12	N/A	38.2	10.8
13–17	N/A	5.2	9.7
18 and more	N/A	57.0	79.5
Year of arrival to Sweden			
Before 1980	N/A	26.7	3.8
1980–1989	N/A	21.0	14.0
1990–1999	N/A	26.3	44.5
2000–2012	N/A	26.0	38.0

Frequencies are expressed as percents, unless otherwise specified. N/A = not applicable.

We assessed linear trends in the prevalence of smoking among immigrants from various origins according to age at arrival by obtaining *p*-values for trends in binomial proportions.

Because of evidence that the timing of immigration in the life cycle affects future life chances^12^ and smoking during pregnancy,^18^ we conducted secondary analyses to explore whether smoking during pregnancy was jointly influenced by age at migration and the birthplace of the father of the child. We included an interaction term and obtained odds ratios with 95% confidence interval for having a Swedish-born partner within strata of age at arrival (arrived during childhood: 0–12 years, during adolescence: 13–17 years, and during adulthood: 18 years and more), before and after adjusting for control variables. In sensitivity analyses, we modified the age at arrival groupings (0–9 years, 10–19 years, and 20 and more years). All analyses were performed using Stata, version 14, software (StatCorp, LP, College Station, TX).

## Results

### Descriptives

There were 2 239 806 pregnancies resulting in a live birth or stillbirth in Sweden in the study period. Of these, we first excluded those of Swedish-born women coupled with a foreign-born father (*n* = 133 845) and foreign-born women coupled with a foreign-born father of a different background than their own (*n* = 64 587). Of the 2 041 374 remaining eligible pregnancies, 259 206 (12.7%) were excluded for one or more of the following reasons: unknown or not classifiable maternal (*n* = 7680) or paternal country of birth (*n* = 118 958), year of immigration or age at arrival (*n* = 17 070), unknown age (*n* = 8), unknown smoking status (*n* = 114 454), and adoptee mothers (*n* = 1036). The final population for analyses included 1 815 026 pregnancies, 324 977 (17.9%) to immigrant women.

Table 1 summarizes women’s sociodemographic characteristics by the type of union they were in. The prevalence of smoking was 11.6%, 11.0%, and 8.9% among Swedish women, foreign-born women in mixed unions, and among women in dual same-origin foreign-born unions, respectively. Foreign-born women lived in socioeconomically disadvantaged households, particularly those in dual same-origin foreign-born unions, most of whom had incomes in the bottom quintile of the income distribution and tended to concentrate in urban areas. Most foreign-born women arrived after adolescence. However, arriving during childhood (0–12 years) was more common among those who had a Swedish partner (38.2%) than among those whose partner was born in the same world region of origin (10.8%). Women in dual same-origin foreign-born unions were also more likely to have migrated to Sweden after 1990s than those in mixed unions.

### Partner’s Birthplace and Smoking During Pregnancy Among Foreign-Born Women

Table 2 presents variations in the smoking rates of immigrant women from different world regions by age at arrival, along with the percentage of immigrant women with a Swedish-born partner. Smoking rates differed according to age at arrival, being generally higher among those who arrived as minors (<18 years) and lowest among those who arrived as adults. For women from most origins, having a Swedish partner was most common among women who immigrated as minors and least common among those who immigrated as adults. Using a different classification of age at arrival groups did not alter these results.

**Table 2.** Number of Pregnancies, Prevalence of Smoking During Pregnancy, and Percentage of Pregnancies With a Swedish-Born Partner, by Women’s Birthplace and Age at Arrival to Sweden, Swedish Pregnancies, 1991–2012

	Number of pregnancies			Smoking during pregnancy				Swedish-born partner			
Age at arrival	0–12 y	13–17 y	18 + y	0–12 y	13–17 y	≥18 y		0–12 y	13–17 y	≥18 y	
Women’s birthplace	*n*	*n*	*n*	%	%	%	*p* ^a^	%	%	%	*p* ^a^
Other Nordic countries (*n* = 38 690)	15 419	2068	21 203	35.7	44.3	18.2	<.001	23.4	33.1	12.8	<.001
Former Yugoslavia (*n* = 41 410)	7496	5916	27 998	19.3	16.8	18.9	<.001	18.4	12.7	13.3	<.001
Eastern Europe (*n* = 27 007)	3844	1631	21 532	22.8	22.3	11.5	<.001	14.5	15.8	8.6	.0930
Middle East (*n* = 97 038)	12 705	9188	75 145	14.4	10.1	6.3	<.001	10.6	6.8	8.7	.0316
Western Europe (*n* = 15 524)	3418	505	11 601	20.5	17.5	5.4	<.001	13.4	13.4	5.8	<.001
Latin America (*n* = 18 577)	6758	1680	10 139	8.0	7.2	5.8	<.001	8.1	5.7	3.7	<.001
United States, Australia, New Zealand, and Israel (*n* = 4217)	921	75	3221	13.3	0.0	3.5	.088	13.5	19.4	4.4	<.001
Sub-Saharan Africa (*n* = 8697)	990	869	6838	12.1	3.5	1.2	<.001	10.8	8.4	6.5	<.001
East Africa (*n* = 25 413)	1160	2465	21 788	7.3	3.8	1.3	<.001	12.9	15.8	8.8	<.001
North Africa (*n* = 6641)	215	175	6251	4.1	4.7	1.4	<.001	14.9	30.8	5.3	<.001
Asia (*n* = 41 763)	12 334	1903	27 526	3.9	1.9	0.9	<.001	11.2	8.2	3.7	<.001

^a^Test for trends for binomial proportions.

Table 3 indicates that women from other Nordic, Former Yugoslavia, and Eastern European countries had lower smoking rates if coupled with a Swedish partner rather than a partner from the same origin. Conversely, having a Swedish-born partner was associated with higher smoking rates among women from the remaining groups, with the only exception of those from Western Europe and United States, Australia, New Zealand and Israel (of whom almost 90% were from the United States), for whom there was no association with the partner’s birthplace, after adjustment.

**Table 3.** Associations Between Having a Native-Born Partner and Smoking During Pregnancy Among Immigrant Women, by Maternal Birthplace, Swedish Pregnancies, 1991–2012

	Partner’s birthplace				Swedish-born partner vs. same as woman’s	
Women’s birthplace	Same as woman’s		Swedish born			
	*N*	Smoking (%)	*N*	Smoking (%)	OR (95% CI)	AOR (95% CI)^a^
Other Nordic countries (*n* = 38 690)	8701	23.2	29 989	18.7	0.76 (0.72, 0.81)	0.72 (0.67, 0.76)
Former Yugoslavia (*n* = 41 410)	36 806	18.7	4604	15.1	0.78 (0.71, 0.84)	0.85 (0.78, 0.93)
Eastern Europe (*n* = 27 007)	13 893	12.8	13 114	10.3	0.79 (0.73, 0.85)	0.87 (0.80, 0.95)
Middle East (*n* = 97 038)	90 820	7.5	6218	9.3	1.26 (1.16, 1.38)	1.19 (1.07, 1.31)
Western Europe (*n* = 15 524)	4479	6.8	11 045	8.2	1.23 (1.07, 1.40)	1.04 (0.63, 1.72)
Latin America (*n* = 18 577)	7419	6.5	11 158	5.8	0.89 (0.79, 1.01)	1.17 (1.02, 1.35)
United States, Australia, New Zealand, and Israel (*n* = 4217)	447	3.8	3770	6.6	1.80 (1.09, 2.97)	1.31 (0.77, 2.24)
Sub-Saharan Africa (*n* = 8697)	6029	1.9	2668	7.7	4.30 (3.41, 5.43)	3.93 (2.99, 5.17)
East Africa (*n* = 25 413)	24 358	1.7	1055	11.6	7.54 (6.10, 9.33)	4.74 (3.56, 6.31)
North Africa (*n* = 6641)	5976	1.6	665	7.7	5.25 (3.70, 7.47)	3.97 (2.57, 6.13)
Asia (*n* = 41 763)	14 962	1.3	26 801	6.8	5.64 (4.85, 6.55)	5.88 (4.98, 6.94)

AOR = adjusted odds ratio; CI = confidence interval; OR = odds ratio.

^a^Adjusted for maternal age groups, paternal age, household income (quintiles), rural residence, cohabitation status, parity, year of arrival to Sweden, and age at arrival to Sweden.

**Figure 1.**
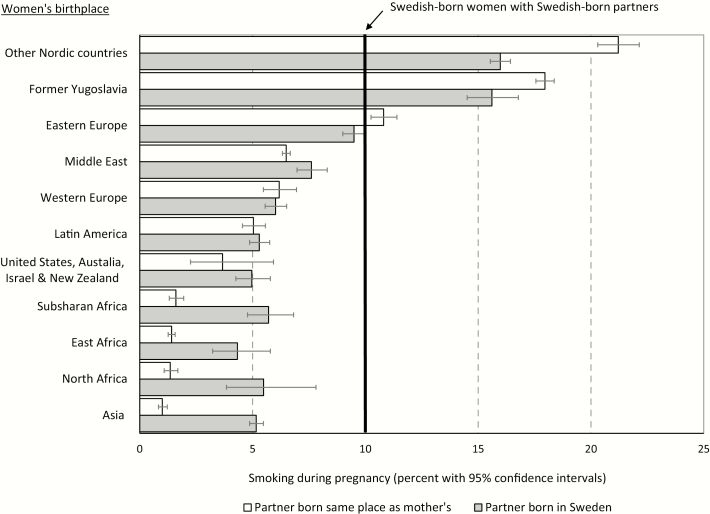
Adjusted prevalence of smoking during pregnancy with 95% confidence intervals, by women’s and partners’ birthplace, Swedish pregnancies, 1991–2012. Prevalence of smoking adjusted for maternal age groups, paternal age, household income (quintiles), rural residence, cohabitation status, parity, year of arrival to Sweden, and age at arrival to Sweden. All differences in the smoking prevalence between foreign-born women with foreign-born partners versus foreign-born women with Swedish-born partners are statistically significant at *p* < .001, with the exception of those from Western Europe, United States, Australia, New Zealand and Israel, and Latin America.

Figure 1 provides a graphic representation of the information contained in Table 2, but expressed in prevalence rates and including a dual Swedish-born union benchmark. Adjusted smoking rates among women in dual foreign-born unions varied by world region of origin (Black squares sorted in descending order); from a high of 21.2% among those from other Nordic countries to a low of 1.0% among women from Asian countries. Swedish-born women sat around the middle of the continuum of risk with a smoking prevalence of 10.2% (vertical line). For women in dual same-origin immigrant unions with smoking rates higher than those in dual Swedish-born unions (other Nordic countries, former Yugoslavia and Eastern Europe), the presence of a Swedish-born partner was associated with reduced smoking rates. Conversely, among women in dual same-origin foreign-born unions with smoking rates lower than those in dual Swedish-born unions, the presence of a Swedish born partner coexisted with higher smoking rates, with the only exception of Western European women.

### Effect Modification by Age at Arrival

Analyses stratified by women’s life stage at immigration (see Supplementary Table S1 online) indicate that having a native-born partner was associated with lower smoking rates among women in high smoking groups (other Nordic countries, former Yugoslavia, and Eastern Europe), particularly among those arrived in adulthood. However, age at arrival significantly modified the association between having a native-born partner and smoking in four immigrant groups: Middle East, Sub-Saharan Africa, East Africa, and Asia (*p*-value for interaction < .001). Among these groups, characterized by lower smoking rates than in the Swedish mainstream population, having a native-born partner was associated with higher smoking rates. The associations were stronger among those who arrived during adolescence and adulthood, with the only exception of women from Middle Eastern countries.

## Discussion

### Main Findings

Having a Swedish-born partner was associated with smoking among immigrant pregnant women; however, the association varied according to women’s birthplace. Among immigrant groups with pregnancy smoking rates higher than those among women in dual Swedish-born unions (other Nordic countries and former Yugoslavia, where rates of smoking in pregnancy are above 20%),^4^ having a Swedish-born partner was associated with reduced smoking rates, whereas for immigrant groups with smoking rates lower than that of women in dual Swedish-born unions (Africa and Asia, regions where smoking during pregnancy is around 1%),^4^ having a Swedish-born partner was associated with much higher smoking rates. This suggests that the presence of a Swedish partner reflects a process of convergence of smoking rates toward the Swedish average.

Pregnancy smoking rates of immigrant women tended to decrease with increasing age at immigration, being lowest among those who immigrated as adults. However, the association between having a Swedish-born partner and smoking differed according to age at arrival for some immigrant groups. Among women from the Middle East, Sub-Saharan Africa, East Africa, and Asia, having a Swedish-born partner resulted in greater increases in smoking prevalence if they arrived during adulthood than during childhood. Conversely, among those from European origins, the influence of a Swedish-born partner did not vary according to age at arrival.

### Limitations

Our study has limitations. First, the lack of information on the partner’s smoking patterns and smoking initiation in relation to couple’s formation prevents determining whether an immigrant woman adopted (or ceased) smoking after her partner’s or chose a partner who shared her already established smoking preferences. This limitation prevents, among other things, identifying the mechanisms through which intermarriage is associated with smoking patterns (acculturation or assortative mating). Furthermore, the lack of this information does not permit us to identify whether acculturation operates through the adoption of the partner’s smoking habits or the adoption of habits that are more predominant in the socioeconomic context to which the couple are exposed. In line with this, a study conducted with the same data showed that smoking during pregnancy among migrant women increases with the time spent in Sweden to the level of Swedish-born women with lower education and income.^19^ Although intermarriage was not considered in that study, it might well occur that migrant women tend to cohabit with Swedish partners with a low socioeconomic condition for whom smoking is more common.^19^ Duration of residence was not the focus of our study but was to some extent accounted for by including age at arrival and age at delivery in the same model. Second, data on other forms of tobacco consumption, such as snus use^20^ or vaping,^21^ were not available for the whole study period. However, their harmful effects are unclear^22,23^ or understudied,^24^ and the prevalence among migrant women is unknown. Third, some degree of selection bias is possible due to >10% of pregnancies being excluded due to missing exposure or outcome data. Fourth, only women who delivered a livebirth or stillbirth were included. Pregnancies that resulted in miscarriages or terminations were not. This may result in a slight underestimation of smoking during pregnancy, as these pregnancy outcomes are associated with smoking.^25^ Fifth, our retrospective study period 1991–2012 may not accurately reflect current prevalence of smoking, although it aligns remarkably well with 2015 estimates for the different immigrant groups and the Swedish.^4^ However, we do not have reasons to believe that the role of partners would be substantially affected. Sixth, our findings may not be generalizable to nonbinary unions. Finally, residual confounding is always possible in observational studies due to the potential influence of mismeasrued or unmeasured factors, such as education. Classification of countries was available in pre-established groups for fathers, which limited the ability to group women in a different way. Year of arrival, used to calculate age at arrival, refers to the year they were granted their residence permit and not when the person actually arrived in the country, which may slightly overestimate age at arrival among asylum seekers later admitted as refugees.

Despite the above-mentioned limitations, this is among the first studies to assess the role of the partner’s origin in smoking habits during pregnancy among migrant women. Furthermore, this study uses multiethnic and nationally representative information based on high-quality registers that have been accurately linked by the Swedish authorities using an exclusive identification number, and for which most control variables are not subject to self-reported issues, nonresponse or interview effects. Furthermore, information on smoking, though self-reported, has been shown to have high agreement in validations studies.^17^

### Interpretation

Our first finding that having a Swedish partner was associated with reduced smoking rates among immigrant women with smoking rates higher than the Swedish and increased smoking rates among those with smoking rates lower than the Swedish represents a “regression to the mean” phenomenon. In both cases, having a native-born partner “pulls” pregnancy smoking rates toward the level observed among women in mainstream dual Swedish-born unions, which smoking rates sit around the middle of the prevalence spectrum. Therefore, the influence of a Swedish partner is not absolute (increasing or decreasing) but rather relative to the prevalence and gendered smoking patterns of the sources countries^26,27^ and reflected among women in same-origin unions. Based on the lack of longitudinal information on smoking initiation and cessation of both partners in relation to the time of immigration, our findings may reflect either the acculturation influence of the native-born partner or a self-selection of immigrant women into marital unions in which the partner holds similar health behaviors. Either way, the partner’s origin seems to be a good marker of assimilation and a relevant source of within-origin variation. Our findings are consistent with previous research documenting associations between smoking and indicators of acculturation, such as generational status,^14^ time since migration^8,19^ and convergence of immigrant smoking patterns with those of the mainstream local population.^28^ Fewer studies explored the potential role of partners. In a study based on Massachusetts’ birth certificates^10^ foreign-born women with US-White partners exhibited five times the odds of smoking during pregnancy than foreign-born women with foreign-born partners. This study did not disaggregate the association by immigrants’ origin nor did account for age at and time from immigration to pregnancy.

We found that having a Swedish-born partner is associated with lower smoking rates among immigrants from regions where women smoke more than Swedish women, but with higher smoking rates among immigrants from regions where women smoke less. This has implications for smoking control strategies. Among immigrant women from countries where women smoke less (e.g., Africa and Asia), primary prevention should be emphasized to prevent them adopting the habit in first place. Family-based interventions may also include the partner to reinforce, if the partner does not smoke, or provide cessation aids, if the partner smokes. However, among immigrant women from countries where women smoke more than the Swedish (e.g., other European countries), a stronger focus should be placed on smoking cessation. While the need for culturally tailored interventions is well established,^5,6,29–31^ immigrants with a native-born partner are more likely to be fluent in the native language and may not require language-specific educational campaigns, unlike immigrant couples from the same geographic origin. All in all, our findings suggest that both maternal and paternal geographic origin are relevant to understand smoking behaviors among immigrant pregnant women and that the epidemiology of smoking should pay greater attention to partners.

Our second interrelated finding is that the association between intermarriage and pregnancy smoking differed according to age at arrival. For some immigrant groups, the presence of a native-born partner may play a greater assimilation influence on women whose socialization occurred in societies that are more culturally distant from that of the western countries, such as those of the Middle East, Sub-Saharan Africa, and Asia, characterized by low smoking rates, particularly among women.^12,27^ Among women in these groups, smoking prevalence decreased with increasing age at arrival, particularly among women in dual same-origin foreign-born unions, which may reflect different degrees of socialization in Sweden by both partners. Lower smoking rates among those arrived at adulthood may reflect women’s smoking habits acquired in their countries of origin whereas the higher rates among those arrived during childhood suggest that smoking initiation took place in Sweden. Overall, these findings shed light on the importance of considering smoking habits among migrants in relation to their smoking patterns in origin while adopting a life course perspective in the receiving context. Though the lack of individual information in origin is a general limitation in migration studies, age at arrival could be used as an instrument to evaluate the relative impact of the country of origin and the assimilation in the host context. Future work that incorporates partners’ smoking habits would provide a more detailed picture of their influence on pregnancy smoking, especially if smoking information, including smoking initiation, is collected before and after couple’s formation. Additional research is needed to explore the role of immigrant partners on the smoking behaviors of native-born women.

## Conclusions

Partners’ birthplace is associated with smoking among immigrant pregnant women. Having a native-born partner exhibits a “regression to the mean” pattern, by which pregnancy smoking rates of immigrant women tend to be “pulled” toward the level of smoking observed among the native-born population. The influence of a native-born partner was stronger among women who immigrated during adulthood and originated in parts of the world where smoking in pregnancy are among the lowest, such as the Middle East, Africa, and Asia. Prevention strategies should be prioritized among these immigrant women upon arrival to prevent them to acquire the habit, particularly if they are single. The need for culturally tailored interventions is well established. Smoking prevention and cessation strategies for adult immigrant women may benefit from involving their partners. A couple-based smoking cessation intervention is a promising approach, but it requires careful implementation to be effective.^32,33^ Among women immigrating during childhood, the context of early socialization may be more important for smoking than the birthplace of their partners’ later in life, which emphasizes the importance of prevention during childhood.

## Supplementary Material

A Contributorship Form detailing each author’s specific involvement with this content, as well as any supplementary data, are available online at https://academic.oup.com/ntr.

ntaa145_suppl_Supplementary_Table_S1

ntaa145_suppl_Supplementray_Taxonomy_Form
