# Supplementary material for: Smoking During Pregnancy Among Immigrant Women With Same-Origin and Swedish-Born Partners
Source: Nicotine Tob Res. 2020 Aug 9;23(2):349–56. doi: 10.1093/ntr/ntaa145 (PMC7822112; doi:10.1093/ntr/ntaa145)
Supplement: ntaa145_suppl_Supplementary_Table_S1 [file ntaa145_suppl_supplementary_table_s1.docx]

**Supplementary Table S1 online. Prevalence of smoking during pregnancy according to woman’s age at arrival and woman’s and partner’s birthplace and Adjusted Odds Ratios (AOR) for having a Swedish partner vs. a partner whose birthplace is the same as that of the woman, Swedish pregnancies, 1991 to 2012**

|  | **Age at arrival** | | | | | | | | | |
| --- | --- | --- | --- | --- | --- | --- | --- | --- | --- | --- |
|  | **0 to 12 years** | | | **13 to 17 years** | | | **18 and more years** | | |  |
|  | **Partner’s birthplace** | |  | **Partner’s birthplace** | |  | **Partner’s birthplace** | |  |  |
| **Woman’s birthplace** | **Same as woman** | **Swedish** | **Swedish-born partner vs. same as woman’s** | **Same as woman** | **Swedish** | **Swedish-born partner vs. same as woman’s** | **Same as woman** | **Swedish** | **Swedish-born partner vs. same as woman’s** |  |
|  | **Smoking %** | | **AOR** ^a^ **(95% CI)** | **Smoking %** | | **AOR** ^a^ **(95% CI)** | **Smoking %** | | **AOR** ^a^ **(95% CI)** | **p-value** ^b^ |
| Other Nordic countries | 35.7 | 23.4 | 0.67 (0.60, 0.75) | 44.3 | 33.0 | 0.69 (0.55, 0.87) | 18.2 | 12.7 | 0.73 (0.67, 0.80) | 0.36 |
| Former Yugoslavia | 19.3 | 18.4 | 0.99 (0.84, 1.17) | 16.9 | 12.7 | 0.81 (0.62, 1.00) | 18.9 | 13.3 | 0.80 (0.70, 0.91) | 0.46 |
| East Europe | 22.8 | 14.5 | 0.83 (0.66, 1.03) | 22.3 | 15.8 | 0.92 (0.70, 1.21) | 11.5 | 8.6 | 0.88 (0.80, 0.98) | 0.70 |
| Middle East | 14.4 | 10.6 | 1.01 (0.86, 1.17) | 10.1 | 6.8 | 0.74 (0.52, 1.04) | 6.3 | 8.7 | 1.75 (1.52, 2.02) | <0.001 |
| West Europe | 20.5 | 13.4 | 0.84 (0.62, 1.14) | 18.0 | 13.4 | 1.01 (0.49, 2.05) | 5.4 | 5.8 | 0.95 (0.80, 1.14) | 0.60 |
| Latin America | 8.0 | 8.1 | 1.47 (1.18, 1.83) | 7.2 | 5.7 | 1.02 (0.65, 1.60) | 5.8 | 3.7 | 0.92 (0.75, 1.14) | 0.15 |
| Sub-Saharan Africa | 12.1 | 10.8 | 0.94 (0.56, 1.59) | 2.5 | 8.4 | 6.05 (2.52, 14.54) | 1.2 | 6.5 | 6.80 (4.81, 9.61) | <0.001 |
| East Africa | 7.4 | 12.9 | 4.23 (2.39, 7.50) | 3.8 | 15.8 | 5.04 (2.90, 8.79) | 1.3 | 8.8 | 5.49 (3.71, 8.13) | <0.001 |
| North Africa | 4.1 | 15.0 | 3.44 (0.86, 13.67) | 4.7 | 30.8 | 4.91 (0.67, 35.91) | 1.4 | 5.3 | 3.52 (2.12, 5.83) | 0.50 |
| Asia | 3.9 | 11.2 | 3.99 (2.99, 5.32) | 1.9 | 8.2 | 9.28 (5.26, 16.37) | 0.9 | 3.7 | 6.93 (5.56, 8.63) | <0.001 |
| Australia, New Zealand, United States & Israel | 13.3 | 12.5 | 0.89 (0.19,4.23) | s | 19.4 | N/A | 3.5 | 4.1 | 1.22 (0.68, 2.17) | N/A |

AOR: Adjusted Odds Ratio; CI: Confidence Interval; s: suppressed due to counts <5; N/A: not available

^a^ Adjusted for maternal age groups, partner’s age, household income (quintiles), rural residence, cohabitation status, parity and year of arrival to Sweden

^b^ p-value for a product term partner’s birthplace x age at arrival within each woman’s birthplace stratum
